# Blood Levels of S-100 Calcium-Binding Protein B, High-Sensitivity C-Reactive Protein, and Interleukin-6 for Changes in Depressive Symptom Severity after Coronary Artery Bypass Grafting: Prospective Cohort Nested within a Randomized, Controlled Trial

**DOI:** 10.1371/journal.pone.0111110

**Published:** 2014-10-20

**Authors:** Daniel M. Pearlman, Jeremiah R. Brown, Todd A. MacKenzie, Felix Hernandez, Souhel Najjar

**Affiliations:** 1 Neuroinflammation Research Group, Epilepsy Center Division, Department of Neurology, NYU School of Medicine, New York, New York, United States of America; 2 The Dartmouth Institute for Health Policy and Clinical Practice, Audrey and Theodor Geisel School of Medicine at Dartmouth, Hanover, New Hampshire, United States of America; 3 Section of Epidemiology and Biostatistics, Department of Community and Family Medicine, Audrey and Theodor Geisel School of Medicine at Dartmouth, Hanover, New Hampshire, United States of America; 4 Section of Cardiology, Department of Medicine, Audrey and Theodor Geisel School of Medicine at Dartmouth, Hanover, New Hampshire, United States of America; 5 Cardiothoracic Surgery, Eastern Maine Medical Center, Bangor, Maine, United States of America; Emory University, United States of America

## Abstract

**Background:**

Cross-sectional and retrospective studies have associated major depressive disorder with glial activation and injury as well as blood–brain barrier disruption, but these associations have not been assessed prospectively. Here, we aimed to determine the relationship between changes in depressive symptom severity and in blood levels of S-100 calcium-binding protein B (S-100B), high-sensitivity C-reactive protein, and interleukin-6 following an inflammatory challenge.

**Methods:**

Fifty unselected participants were recruited from a randomized, controlled trial comparing coronary artery bypass grafting procedures performed with versus without cardiopulmonary bypass for the risk of neurocognitive decline. Depressive symptom severity was measured at baseline, discharge, and six-month follow-up using the Beck Depression Inventory II (BDI-II). The primary outcome of the present biomarker study was acute change in depressive symptom severity, defined as the intra-subject difference between baseline and discharge BDI-II scores. Blood biomarker levels were determined at baseline and 2 days postoperative.

**Results:**

Changes in S-100B levels correlated positively with acute changes in depressive symptom severity (Spearman ρ, 0.62; P = 0.0004) and accounted for about one-fourth of their observed variance (*R^2^*, 0.23; P = 0.0105). This association remained statistically significant after adjusting for baseline S-100B levels, age, weight, body-mass index, or β-blocker use, but not baseline BDI-II scores (P = 0.064). There was no statistically significant association between the primary outcome and baseline S-100B levels, baseline high-sensitivity C-reactive protein or interleukin-6 levels, or changes in high-sensitivity C-reactive protein or interleukin-6 levels. Among most participants, levels of all three biomarkers were normal at baseline and markedly elevated at 2 days postoperative.

**Conclusions:**

Acute changes in depressive symptom severity were specifically associated with incremental changes in S-100B blood levels, largely independent of covariates associated with either. These findings support the hypothesis that glial activation and injury and blood–brain barrier disruption can be mechanistically linked to acute exacerbation of depressive symptoms in some individuals.

## Introduction

A large body of evidence substantiates the association between inflammation and major depressive disorder [1]–[13]. Cross-sectional and retrospective studies of depression have documented microglial activation and proliferation [3], [4], [14] as well as astroglial loss in biopsied and post-mortem brain tissue [15]–[18], and increased Th1 proinflammatory cytokines in cerebrospinal fluid (CSF) and blood samples [6]–[12] (recently reviewed in [1]). In addition, population-level studies identify autoimmune diseases and severe infections as risk factors for depression [19], [20]. Prospective etiologic [5], [21]–[31] and treatment studies [3], [32]–[35] have provided some of the most direct evidence supporting the hypothesis that inflammation can be causally related to depressive symptoms. The prototypical etiologic studies in this regard are the so-called inflammation-challenge paradigms, such as interferon-α administration [36]. Among those treated with interferon-α, typically for hepatitis C or cancer, about half experience acute-onset depression in association with systemic inflammatory and neuroimaging abnormalities [5], [21]–[31]. These inflammation-challenge paradigms have also been extended to include orthopedic surgery [37], and more recently–cardiac surgery [38], [39], in which the occurrence of a robust systemic inflammatory response is well-established [40].

A related and emerging area of research in psychoimmunology has focused on elucidating the connections among inflammatory abnormalities as well as glial activation and injury and blood–brain barrier disruption in depression [2], [3]. Like inflammation, evidence of glial activation and injury in depression has been extensively documented [1]. Despite this, however, and notwithstanding the necessary functions of astroglial–endothelial interactions in maintaining blood–brain barrier integrity [35], only recently has evidence linking blood–brain barrier disruption to depression begun to accumulate [2], [3]. Evidence to this effect includes histological and ultrastructural findings in biopsied and post-mortem brain tissue [3], [41]; elevated CSF-to-serum albumin ratios [42]–[44]; reduced flow-mediated dilation (a surrogate of endothelial dysfunction) [45]; increased vascular disease comorbidity [46], [47]; and elevated blood levels of endothelial dysfunction biomarkers (e.g., intercellular adhesion molecule 1, vascular adhesion molecule 1, E-selectin, and P-selectin) [48]–[61].

S-100 calcium-binding protein B (S-100B) is a 10 kDa protein, consisting of 92 amino acids and two EF-hand domains, that is implicated in the pathophysiology of major depressive disorder [55], [59], [62]–[71] and various neurological disorders [72]–[74]. S-100B is produced and secreted by both intracerebral (i.e., mainly astrocytes, and to a lesser degree, oligodendrocytes) and extracerebral sources (e.g., adipocytes and myocytes) [72], and is involved in numerous regulatory and immune pathways [75], [76]. Elevated blood S-100B levels are considered a marker of glial activation and injury [72], [74], [77]–[87] as well as blood–brain barrier disruption [72], [81], [88]–[93], and have been associated with suicidality [56], lower Glasgow Coma Scale scores [73], greater post-concussive symptom severity [74], ictal events [72], and greater risk of postoperative cognitive decline [94]–[97] (in addition to numerous other clinically significant outcomes). To date, however, no studies have simultaneously assessed the relationships between depression and glial activation and injury, blood–brain barrier disruption, and inflammation using a prospective design. Here, we aimed to address this gap in the literature by evaluating perioperative changes in blood levels of S-100B, high-sensitivity C-reactive protein (hs-CRP), and interleukin-6 for changes in depressive symptom severity in the inflammation-challenge paradigm of cardiac surgery.

## Materials and Methods

### Design, setting, and participants

This is a prospective cohort study nested within a randomized, controlled trial. An unselected group of 50 participants was recruited from among 201 patients undergoing coronary artery bypass grafting in the context of a randomized, controlled trial comparing conventional vs off-pump procedures for the risk of neurocognitive decline (Scarecrow trial, 2001–2004) [98]. Inclusion criteria were ages 40 to 80 years, clinical indication for urgent or elective coronary artery bypass grafting, referral to the Cardiothoracic Surgery service at Eastern Maine Medical Center (Bangor, Maine, United States), and capacity to provide written informed consent. Exclusion criteria were concomitant surgical procedures, severe calcification of the ascending aorta or deep intra-myocardial left anterior descending coronary artery, recent administration of an inotropic agent in excess of 3 µg/kg/min, requiring a cardiac-assist device for hemodynamic instability, and not providing written informed consent. Details of the surgical and anesthesia protocols used in the trial have been described previously [98]. All participants provided written informed consent. The Institutional Review Board of Eastern Maine Medical Center approved this study.

### Biochemical measurements

Blood samples were collected by standard venipuncture at baseline and approximately 2 days postoperative. Upon collection, samples were stored at −80°C and then transferred on dry ice to the Laboratory for Clinical and Biochemical Research (Colchester, Vermont, United States) where S-100B, hs-CRP, and interleukin-6 blood levels were determined in either plasma or serum. Enzyme-linked immunosorbent assays were used to determine S-100B (#364701D, DiaSorin, Bromma, Sweden) and interleukin-6 blood levels (#HS600B, R&D Systems, Minnesota, United States). An automated BNII nephelometer was used to perform an immunonephelometric assay to determine hs-CRP blood levels (Siemens Healthcare Diagnostics, Illinois, United States). All laboratory personnel were blinded to participant characteristics and clinical information.

Lower limits of detection were 0.02 µg/l for S-100B, 0.16 mg/l for hs-CRP, and 0.16 pg/ml for interleukin-6. Inter-assay coefficients of variance at baseline and 2 days postoperative were 6.01% and 3.31% for S-100B, 2.93% and 1.82% for hs-CRP, and 7.95% and 8.71% for interleukin-6. In determining S-100B and interleukin-6 blood levels, each sample was run twice, the arithmetic mean of which was taken as the final value. In determining hs-CRP blood levels, each sample was run once, as the assays were automated. The approximate upper limits of normal blood levels for each biomarker are 0.15 µg/l for S-100B, 10.0 mg/l for hs-CRP, and 5.0 pg/ml for interleukin-6. hs-CRP levels less than or equal to 1.0 mg/l are indicative of low risk for ischemic heart disease, 1.0 to 3.0 mg/l moderate risk, and 3.0 to 10.0 mg/l high risk. hs-CRP levels greater than or equal to 10.0 mg/l are indicative of acute systemic inflammation of a non-cardiac origin (e.g., as occurs with infection or cardiac surgery). The change (i.e., intra-subject difference) between blood levels of each biomarker at baseline and 2 days postoperative was computed for each participant. Instances where 2-day postoperative blood levels were greater than baseline levels (for a given biomarker and participant), were considered a positive change (i.e., perioperative increases). Likewise, when 2-day postoperative blood levels were less than baseline levels, this was considered a negative change (i.e., perioperative decreases).

### Main outcomes

Depressive symptom severity was assessed at baseline, discharge, and six-month month follow-up using the Beck Depression Inventory II (BDI-II). Scores ranging from 0 to 13 indicate subclinical or minimal depressive symptom severity, 14 to 19 mild, 20 to 28 moderate, and 29 to 63 severe [99]. Participants, family members, treating clinicians, BDI-II test examiners, and all other trial staff in contact with participants were blinded to the results of blood biomarker levels. The primary outcome of the present biomarker study, acute change in depressive symptom severity, was defined as the intra-subject difference between baseline and discharge BDI-II scores. The secondary outcome, delayed change in depressive symptom severity, was defined as the intra-subject difference between baseline and six-month follow-up BDI-II scores.

### Statistical analysis

Analyses were done using Stata version 12.1 for Mac (StataCorp, LP. College Station, Texas, United States). Participant characteristics were summarized as the arithmetic mean ± standard deviation for continuous data and frequency and percentage for categorical data. These descriptive statistics were then compared between the biomarker and non-biomarker cohorts that comprised the Scarecrow trial with the use of two-tailed Student *t* tests for continuous data and Cochran–Mantel–Haenszel *χ^2^* tests for categorical data. To identify covariates of blood biomarker measures at baseline and their differences between baseline and postoperative assessments and/or the main outcomes, Spearman rank correlation coefficients (Spearman ρ) were calculated for all possible two-term combinations, applying the pairwise deletion method for comparisons involving missing data. Participant characteristics that yielded statistically significant correlations were entered into the relevant multiple linear regression models (see below).

Spearman ρ values were calculated and scatterplots generated for all possible two-term combinations between and among blood biomarker measures at baseline and postoperative assessments, and the difference between them and BDI-II scores at baseline, discharge, six-month follow-up, the difference between baseline and discharge BDI-II scores (primary outcome), and the difference between baseline and six-month follow-up BDI-II scores (secondary outcome). For statistically significant correlations between blood biomarker measures and the main outcomes, we did sensitivity analysis by recalculating Spearman ρ values using Bonferroni-adjusted significance levels and again using Sidak-adjusted significance levels. Last, the main outcomes were regressed on blood biomarker measures at baseline and the difference between blood biomarker levels at baseline and postoperative assessments using ordinary-least squares simple and multiple linear regressions. Before entering terms into the models, variable transformations were first applied to all variables that did not have a normal distribution. The transformation used for a given variable was based on the lowest *χ^2^* value yielded by a ladder of powers analysis [100].

## Results

### Biomarker cohort

**Figure 1:**
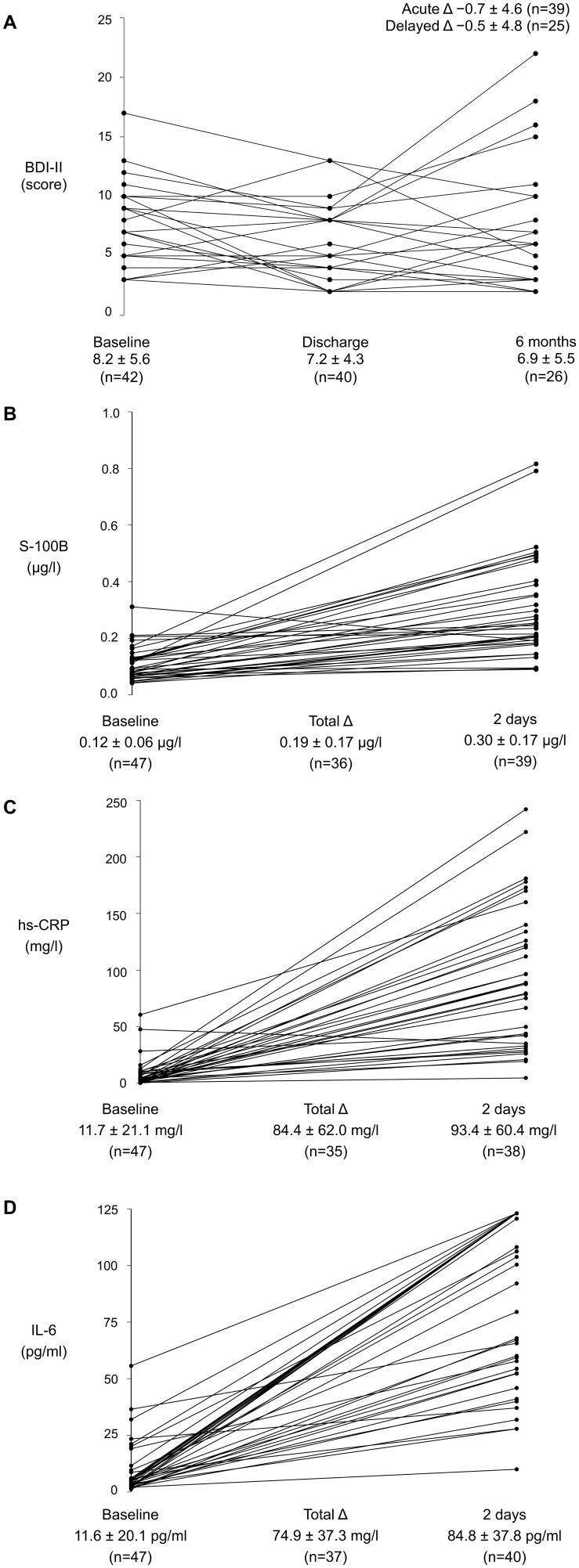
Paired distributions of Beck Depression Inventory II scores and blood biomarker measures. (A) Beck Depression Inventory II scores at baseline, discharge, six-month follow-up; (B) S-100 calcium-binding protein B levels at baseline, 2 days postoperative, and the total change between them; (C) high-sensitivity C-reactive protein levels at baseline 2 days postoperative, and the total change between them; (D) inteleukin-6 levels at baseline 2 days postoperative, and the total change between them. Values given below the x-axis are arithmetic mean ± standard deviation. BDI-II = Beck Depression Inventory II. hs-CRP = high-sensitivity C-reactive protein. IL-6 = interleukin-6. S-100B = S-100 calcium-binding protein B.

The pairwise distributions of BDI-II scores and blood biomarker levels are displayed in Figure 1. Participant characteristics and BDI-II scores were generally similar among those comprising the biomarker cohort relative to the remaining participants in the Scarecrow trial (Table 1). One exception was discharge BDI-II scores, which were lower on average among those in the biomarker cohort (Table 1).

**Table 1:** Prospective biomarker cohort, nested within the Scarecrow trial (2001–2004).

	Biomarker cohort	Non-biomarker cohort	P Value*
	(n = 50)	(n = 151)	
Age (years)	63.0±8.3	64.2±9.5	0.43
Female	6 (12.0)	34 (22.5)	0.11
Weight (kg)	87.7±12.9	88.3±14.9	0.83
Height (cm)	172.5±9.3	172.8±8.8	0.87
Body-mass index (kg/m^2^)	29.5±3.8	29.5±4.2	0.95
History of vascular disease	8 (16.0)	34 (22.5)	0.33
History of diabetes mellitus	17 (34.0)	49 (32.5)	0.84
History of COPD	3 (6.0)	13 (8.6)	0.56
History of smoking	10 (20.0)	28 (18.5)	0.82
Charlson comorbidity index	0.62±0.70	0.77±0.92	0.28
Myocardial infarction†	8 (16.0)	17 (11.3)	0.38
Unstable angina at admission	35 (70.0)	88 (58.3)	0.14
Two-vessel disease	18 (36.0)	43 (28.5)	0.32
Three-vessel disease	27 (54.0)	85 (56.3)	0.78
Left-ventricular ejection fraction (%)‡	61.4±10.8	58.9±13.3	0.25
Troponin T at 24 hours (ng/dl)	0.62±0.61	0.58±1.01	0.77
NYHA class (I-IV)§	1.70±0.83	1.75±0.85	0.75
Serum creatinine (mg/dl)	1.05±0.24	1.15±0.73	0.33
β-blocker	39 (78.0)	112 (74.2)	0.59
ACE-inhibitor	11 (22.0)	49 (32.5)	0.16
Ca^2+^ channel blocker	11 (22.0)	24 (15.9)	0.32
Acetylsalicylic acid†	39 (78.0)	106 (70.2)	0.29
Randomized to off-pump procedure	23 (46.0)	76 (50.3)	0.60
Underwent off-pump procedure	21 (42.0)	70 (46.4)	0.59
Urgent priority procedure	30 (60.0)	95 (62.9)	0.71
Time from baseline to procedure (days)	3.1±3.6	2.6±3.3	0.34
Time from procedure to discharge (days)	6.0±2.6	7.3±4.9	0.08
BDI-II (score), baseline (n = 42; n = 145)	8.2±5.6	9.6±6.9	0.23
BDI-II (score), discharge (n = 40; n = 121)	7.2±4.3	9.8±6.7	0.026
BDI-II (score), 6 months (n = 26; n = 105)	6.9±5.5	7.8±7.4	0.59
BDI-II (score), acute Δ (n = 39; n = 120)||	−0.7±4.6	0.0±5.4	0.43
BDI-II (score), delayed Δ (n = 25; n = 104)¶	−0.5±4.8	−1.8±5.7	0.29

Values are arithmetic mean ± standard deviation for continuous data, and frequency (percentage) for categorical data. The Scarecrow trial compared CABG performed with vs. without cardiopulmonary bypass for the risk of postoperative neurocognitive decline (defined as a ≥20% reduction on ≥20% of 19 psychometrics) among 201 patients at Eastern Maine Medical Center. Abbreviations: ACE = angiotensin-converting enzyme. CABG = coronary artery bypass grafting. COPD = chronic obstructive pulmonary disease. hs-CRP = high-sensitivity C-reactive protein. NYHA = New York Heart Association. S-100B = S-100 calcium-binding protein B.

*Two-tailed Student *t* test for continuous data; *χ^2^* test for categorical data.

† Within 7 days before the index procedure.

‡ Missing data for: n = 3; n = 19.

§ Missing data for: n = 3; n = 3.

|| Acute Δ refers to the primary outcome, acute change in depressive symptom severity, defined as the intra-subject difference between BDI-II scores at baseline and discharge.

¶ Delayed Δ refers to the secondary outcome, delayed change in depressive symptom severity, defined as the intra-subject difference between BDI-II scores at baseline and six-month follow-up.

### Covariate assessment

**Figure 2:**
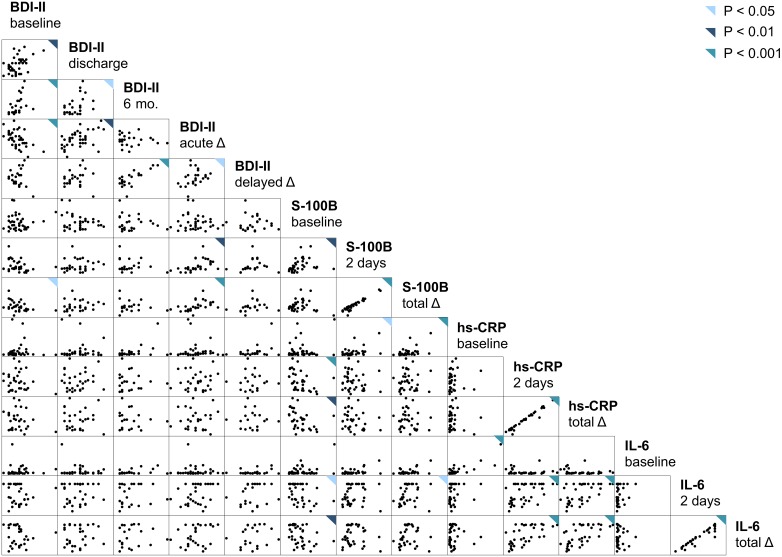
Scatterplot matrix of all possible combinations between and among Beck Depression Inventory II scores and blood biomarker measures. BDI-II scores were assessed at baseline, discharge, and 6 months. Differences between baseline and discharge BDI-II scores (primary outcome) and baseline and 6-month BDI-II scores (secondary outcome) were computed for each participant. Biomarker levels were determined at baseline and 2 days postoperatively, and change scores computed. BDI-II = Beck Depression Inventory II. hs-CRP = high-sensitivity C-reactive protein. IL-6 = interleukin-6. S-100B = S-100 calcium-binding protein B.

Spearman ρ values for comparisons between participant characteristics and the main outcomes and blood biomarker measures are displayed in Table 2. None of the participant characteristics showed a statistically significant correlation with either outcome (Table 2). Intra-subject differences between baseline and postoperative S-100B levels correlated positively with age and negatively with weight and body-mass index, and were significantly lower on average among those taking β-blockers (Table 2). Perioperative increases in hs-CRP levels correlated negatively with age. Perioperative increases in hs-CRP and interleukin-6 levels were significantly greater on average among participants randomized to (intention-to-treat) and having undergone (as-treated) off-pump procedures, as compared with those randomized to and having undergone conventional procedures (Table 2). Spearman ρ values and scatterplots for all possible two-term combinations between and among BDI-II scores and blood biomarker measures are displayed in Table 3 and Figure 2.

**Table 2:** Spearman rank correlation coefficients for comparisons between participant characteristics and main outcomes as well as blood biomarker levels.

	BDI-II (score)		S-100B (µg/l)		hs-CRP (mg/l)		IL-6 (pg/ml)	
	Acute Δ	Delayed Δ	Baseline	Total Δ	Baseline	Total Δ	Baseline	Total Δ
	(n = 39)†	(n = 25)‡	(n = 47)	(n = 36)§	(n = 47)	(n = 35)§	(n = 47)	(n = 37)§
Age (years)	0.30	0.04	0.23	0.39*	0.15	–0.35*	0.23	–0.22
Female	0.07	0.05	0.14	0.16	0.08	0.13	0.08	0.12
Weight (kg)	–0.14	–0.05	–0.21	–0.38*	–0.04	0.07	0.00	0.16
Height (cm)	–0.01	0.15	–0.12	–0.06	–0.07	0.10	–0.01	0.10
Body-mass index (kg/m^2^)	–0.22	–0.21	–0.15	–0.34*	0.05	–0.01	0.06	0.06
History of vascular disease	–0.04	–0.11	0.07	0.10	–0.08	0.12	0.00	0.08
History of diabetes mellitus	0.03	–0.19	–0.16	–0.07	–0.33*	–0.05	–0.05	–0.10
History of COPD	0.04	0.16	0.11	0.20	–0.21	0.04	–0.08	–0.12
History of smoking	–0.06	0.00	–0.11	0.03	0.08	–0.09	0.10	0.12
Charlson comorbidity index	–0.03	–0.14	–0.06	0.16	–0.37*	0.05	–0.05	0.01
Myocardial infarction||	–0.15	0.39	–0.04	–0.10	0.34*	0.14	0.42**	0.24
Unstable angina at admission	0.11	–0.09	–0.08	0.15	0.36*	0.14	0.40**	0.23
Two-vessel disease	0.07	–0.20	–0.01	–0.26	0.07	0.02	0.15	0.13
Three-vessel disease	–0.18	0.22	–0.10	0.26	0.02	0.06	–0.14	0.03
Left-ventricular ejection fraction (%)††	–0.08	–0.06	0.17	–0.23	–0.09	–0.19	–0.24	–0.28
Troponin T at 24 hours (ng/dl)	–0.10	0.02	0.30*	0.21	0.30*	–0.21	0.16	–0.14
NYHA class (I-IV)‡‡	0.04	–0.19	0.21	0.29	–0.14	–0.25	0.04	–0.24
Serum creatinine (mg/dl)§§	0.05	–0.05	0.09	–0.02	0.06	–0.08	0.15	–0.09
β-blocker	–0.05	–0.13	0.07	–0.39*	–0.30*	–0.17	–0.20	–0.15
ACE-inhibitor	–0.21	0.04	–0.21	–0.01	–0.22	0.19	–0.06	0.19
Ca^2+^ channel blocker	0.10	0.23	–0.13	0.15	0.20	0.24	–0.21	0.45**
Acetylsalicylic acid†	0.29	0.01	0.12	0.12	–0.05	–0.30	–0.26	–0.21
Randomized to off-pump procedure	0.21	–0.16	–0.39**	–0.01	–0.01	0.50**	–0.05	0.54***
Underwent off-pump procedure	0.26	–0.06	–0.41**	0.02	–0.07	0.50**	–0.09	0.53***
Urgent priority procedure	0.01	0.24	0.19	0.29	0.23	0.00	0.59***	0.10
Time from baseline to procedure (days)	0.02	0.24	0.18	0.34*	0.19	0.00	0.41**	0.16
Time from procedure to discharge (days)	0.20	–0.15	–0.06	0.09	–0.02	0.14	0.31*	0.05

Pairwise case deletion was used for missing observations. Abbreviations: ACE = angiotensin-converting enzyme. BDI-II = Beck Depression Inventory II. CABG = coronary artery bypass grafting. COPD = chronic obstructive pulmonary disease. hs-CRP = high-sensitivity C-reactive protein. IL-6 = interleukin-6. NYHA = New York Heart Association. S-100B = S-100 calcium-binding protein B.

*P<0.05; **P<0.01; ***P<0.001.

† Acute Δ refers to the primary outcome, acute change in depressive symptom severity, defined as the intra-subject difference between BDI-II scores at baseline and discharge.

‡ Delayed Δ refers to the secondary outcome, delayed change in depressive symptom severity, defined as the intra-subject difference between BDI-II scores at baseline and six-month follow-up.

§ Total Δ refers to the intra-subject difference in blood biomarker levels between baseline and 2 days postoperative.

|| Within 7 days before the index procedure.

†† Missing data (from left to right): n = 3; n = 1; n = 3; n = 2; n = 2; n = 3; n = 2; n = 3; n = 2.

‡‡ Missing data (from left to right): n = 0; n = 1; n = 3; n = 2; n = 2; n = 3; n = 2; n = 3; n = 3.

§§ Missing data (from left to right): n = 0; n = 1; n = 0; n = 0; n = 0; n = 0; n = 0; n = 0; n = 0.

**Table 3:** Spearman rank correlation coefficients for comparisons between and among Beck Depression Inventory II scores and blood biomarker measures.

		BDI-II (score)					S-100B (µg/l)			hs-CRP (mg/l)			IL-6 (pg/ml)		
		Baseline	Discharge	6 months	Acute Δ	Delayed Δ	Baseline	2 days	Total Δ	Baseline	2 days	Total Δ	Baseline	2 days	Total Δ
**BDI-II (score)**	**Baseline**	.	0.46**	0.66***	−0.52***	−0.03	0.02	−0.31	−0.43*	−0.01	−0.01	−0.21	0.24	−0.26	−0.22
		.	39	25	39	25	39	33	30	39	39	29	39	34	31
	**Discharge**	0.46**	.	0.48*	0.46**	0.34	−0.01	0.11	0.18	0.10	0.04	−0.10	0.13	0.06	0.07
		39	.	26	39	25	37	31	28	37	30	27	37	32	29
	**6 months**	0.66***	0.48*	.	−0.14	0.67***	−0.13	−0.06	0.03	0.28	−0.01	−0.12	0.26	0.14	0.13
		25	26	.	25	25	25	22	21	25	21	20	25	23	22
	**Acute Δ**	−0.52***	0.46**	−0.14	.	0.40*	0.02	0.55**	0.62***	0.21	0.05	−0.00	0.06	0.34	0.29
		39	39	25	.	25	36	31	28	36	30	27	36	32	29
	**Delayed Δ**	−0.03	0.34	0.67***	0.40*	.	0.10	0.47*	0.42	0.32	−0.03	−0.07	0.10	0.29	0.26
		25	25	25	25	.	24	22	21	24	21	20	24	23	22
**S-100B (µg/l)**	**Baseline**	0.02	−0.01	−0.13	0.02	0.10	.	0.51**	0.10	−0.09	−0.56***	−0.53**	0.02	0.39*	−0.42**
		39	37	25	36	24	.	36	36	47	35	35	47	37	37
	**2 days**	−0.31	0.11	−0.06	0.55**	0.47*	0.51**	.	0.89***	0.41*	−0.22	−0.25	0.25	0.21	0.07
		33	31	22	31	22	36	.	36	36	38	35	36	39	36
	**Total Δ**	−0.43*	0.18	0.03	0.62***	0.42	0.10	0.89***	.	0.54***	0.02	−0.05	0.28	0.37*	0.24
		30	28	21	28	21	36	36	.	36	35	35	36	36	36
**hs-CRP (mg/l)**	**Baseline**	−0.01	0.10	0.28	0.21	0.32	−0.09	0.41*	0.54***	.	0.23	0.05	0.59***	0.29	0.10
		39	37	25	36	24	47	36	36	.	35	35	47	37	37
	**2 days**	−0.11	0.04	−0.01	0.05	−0.03	−0.56***	−0.22	0.02	0.23	.	0.97***	−0.02	0.52***	0.63***
		32	30	21	30	21	35	38	35	35	.	35	35	38	35
	**Total Δ**	−0.21	−0.10	−0.12	−0.00	−0.07	−0.53**	−0.25	−0.05	0.05	0.97***	.	−0.15	0.54***	0.62***
		29	27	20	27	20	35	35	35	35	35	.	35	35	35
**IL-6 (pg/ml)**	**Baseline**	0.24	0.13	0.26	0.06	0.10	0.02	0.25	0.28	0.59***	−0.02	−0.15	.	0.21	−0.08
		39	37	25	36	24	47	36	36	47	35	35	.	37	37
	**2 days**	−0.26	0.06	0.14	0.34	0.29	0.39*	0.21	0.37*	0.29	0.52***	0.54***	0.21	.	0.93***
		34	32	23	32	23	37	39	36	37	38	35	37	.	37
	**Total Δ**	−0.22	0.07	0.13	0.29	0.26	−0.42**	0.07	0.24	0.10	0.63***	0.62***	−0.08	0.93***	.
		31	29	22	29	22	37	36	36	37	35	35	37	37	.

Values in the cells for each comparison are (from top to bottom): the Spearman rank correlation coefficient (Spearman *ρ*) and number of observations. Pairwise case deletion was used for missing observations. Abbreviations: ACE = angiotensin-converting enzyme. BDI-II = Beck Depression Inventory II. CABG = coronary artery bypass grafting. COPD = chronic obstructive pulmonary disease. hs-CRP = high-sensitivity C-reactive protein. NYHA = New York Heart Association. S-100B = S-100 calcium-binding protein B.

*P<0.05; **P<0.01; ***P<0.001.

† Acute Δ refers to the primary outcome, acute change in depressive symptom severity, defined as the intra-subject difference between BDI-II scores at baseline and discharge.

‡ Delayed Δ refers to the secondary outcome, delayed change in depressive symptom severity, defined as the intra-subject difference between BDI-II scores at baseline and six-month follow-up.

§ Total Δ refers to the intra-subject difference in blood biomarker levels between baseline and 2 days postoperative.

### Acute change in depressive symptom severity

**Figure 3:**
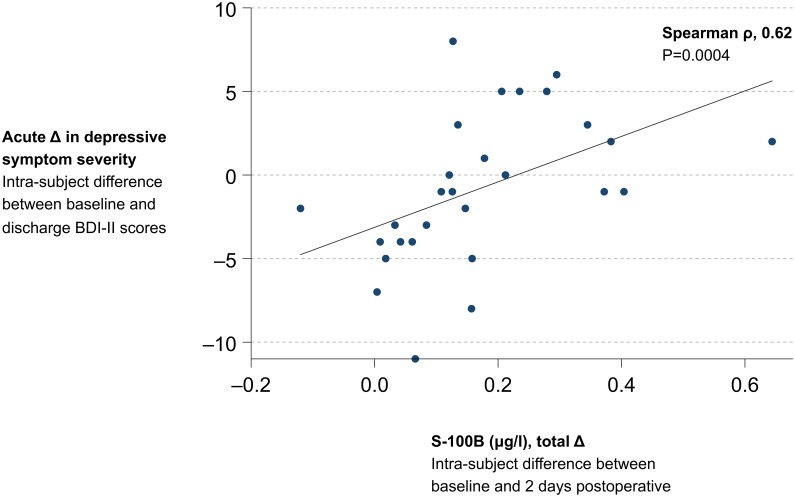
Association between perioperative increases in blood levels of S-100 calcium-binding protein B and acute changes in depressive symptom severity. The x-axis refers to the intra-subject difference between S-100 calcium binding protein B (S-100B) levels measured at baseline and 2 days postoperative. The y-axis refers to the primary outcome, acute change in depressive symptom severity, defined as the intra-subject difference between baseline and discharge Beck Depression inventory II (BDI-II) scores. BDI-II = Beck Depression Inventory II. S-100B = S-100 calcium-binding protein B. Spearman rho = Spearman rank correlation coefficient.

Intra-subject differences between baseline and postoperative S-100B levels exhibited a strong positive correlation with intra-subject differences between baseline and discharge BDI-II scores (Spearman ρ, 0.62; P = 0.0004; Table 3; Figure 3). Neither the strength nor the statistical significance of this correlation was affected by Bonferroni- or Sidak-adjusted significance levels (for both adjustments: Spearman ρ, 0.62; P = 0.0004). There were no other statistically significant correlations between blood biomarker measures and the primary outcome (Table 3). Significant negative correlations were observed between baseline BDI-II scores and changes in S-100B levels and between baseline BDI-II scores and acute changes in depressive symptom severity (Table 3).

The results of simple and multiple linear regressions of the primary outcome on blood biomarker measures are displayed in Table 4. A simple linear regression model showed that each 0.10-µg/l increase in intra-subject differences between baseline and postoperative S-100B levels was associated with a 1.36-point increase in intra-subject differences between baseline and discharge BDI-II scores (regression coefficient [*R*] = 13.60, coefficient of determination [*R^2^*] = 0.23, *t* = 2.76, P = 0.010, standardized regression coefficient [*β*] = 0.48, *F*_(1,26)_ = 7.61, P = 0.0105; Table 4; Figure 3). This association remained statistically significant after individual adjustments for baseline S-100B levels, age, weight, body-mass index, and β-blocker use, but not baseline BDI-II scores (*R* = 9.18, *R^2^* = 0.40, *t* = 1.94, P = 0.064) adjusting for which improved the explanatory power of the model overall (*β* = 0.32, *R^2^*_adjusted_ = 0.35, *F*_(2,25)_ = 8.29, P model = 0.0017; Table 4). There were no statistically significant associations between the primary outcome and the remaining blood biomarker measures (Table 3; Table 4).

**Table 4:** Linear regression of the primary outcome on blood biomarker measures and covariates.

Independent variables		Acute Δ in depressive symptom severity*					
Biomarker measures	Covariates	β	n	R^2^	R^2^_adjusted_	RMSE	P Value†
S-100B (µg/l), baseline	Unadjusted	−0.02	36	0.00	−0.03	4.72	0.92
	BDI-II (scores), baseline	−0.16	36	0.30	0.25	4.02	0.91
	Underwent off-pump procedure	0.22	36	0.15	0.09	4.43	0.07
	Urgent priority procedure	−0.03	36	0.01	−0.05	4.76	0.82
	Troponin T at 24 hours (ng/dl)	0.07	36	0.05	−0.01	4.68	0.47
S-100B (µg/l), total Δ	Unadjusted	0.48	28	0.23	0.20	4.03	0.010
	S-100B (µg/l), baseline	0.47	28	0.24	0.18	4.09	0.012
	BDI-II (scores), baseline	0.32	28	0.40	0.35	3.63	0.06
	Age (years)	0.42	28	0.26	0.20	4.02	0.030
	Weight (kg)	0.40	28	0.25	0.20	4.04	0.046
	Body-mass index (kg/m^2^)	0.44	28	0.23	0.17	4.09	0.025
	β-blocker	0.53	28	0.24	0.18	4.08	0.011
hs-CRP (mg/l), baseline	Unadjusted	0.07	36	0.01	−0.02	4.71	0.67
	BDI-II (scores), baseline	0.08	36	0.30	0.26	4.00	0.58
	Charlson comorbidity index	0.13	36	0.02	−0.04	4.73	0.48
	Myocardial infarction†	0.07	36	0.01	−0.05	4.77	0.69
	Troponin T at 24 hours (ng/dl)	0.12	36	0.05	−0.00	4.66	0.50
hs-CRP (mg/l), total Δ	Unadjusted	0.03	27	0.00	−0.04	4.67	0.87
	hs-CRP (mg/l), baseline	0.05	27	0.09	0.02	4.55	0.79
	BDI-II (scores), baseline	−0.03	27	0.31	0.25	3.96	0.86
	Age (years)	0.15	27	0.13	0.05	4.46	0.47
	Underwent off-pump procedure	−0.17	27	0.06	−0.02	4.63	0.52
	Urgent priority procedure	−0.02	27	0.13	0.06	4.45	0.92
IL-6 (pg/ml), baseline	Unadjusted	−0.05	36	0.00	−0.03	4.71	0.76
	BDI-II (scores), baseline	−0.10	36	0.31	0.26	3.99	0.50
	Myocardial infarction‡	−0.05	36	0.01	−0.05	4.77	0.77
	Unstable angina at admission	0.04	36	0.03	−0.02	4.71	0.82
IL-6 (pg/ml), total Δ	Unadjusted	0.23	29	0.06	0.02	4.37	0.22
	IL-6 (pg/ml), baseline	0.23	29	0.08	0.01	4.41	0.23
	BDI-II (scores), baseline	0.13	29	0.32	0.27	3.77	0.44
	Acetylsalicylic acid‡	0.27	29	0.08	0.01	4.39	0.18
	Underwent off-pump procedure	0.18	29	0.06	−0.01	4.44	0.47
	Urgent priority procedure	0.19	29	0.16	0.09	4.21	0.31

BDI-II = Beck Depression Inventory II. *β* = standardized regression coefficient. hs-CRP = high-sensitivity C-reactive protein. IL-6 = interleukin-6. *R^2^* = coefficient of determination. *R^2^*_adjusted_ = adjusted coefficient of determination. RMSE = root-mean-square error. S-100B = S-100 calcium-binding protein B.

*Acute Δ refers to the primary outcome, acute change in depressive symptom severity, defined as the intra-subject difference between BDI-II scores at baseline and discharge

† Corresponds to the blood biomarker term in the model.

‡ Within 7 days before the index procedure.

### Delayed change in depressive symptom severity

Spearman ρ values and scatterplots for all possible two-term combinations between the secondary outcome and blood biomarker measures are displayed in Table 3 and Figure 2. There were no statistically significant correlations between blood biomarker levels and the secondary outcome (Table 3). The results of simple and multiple linear regressions of the secondary outcome on blood biomarker levels are displayed in Table 5. None of these models, unadjusted or adjusted, yielded a statistically significant relationship between blood biomarker levels and delayed change in depressive symptom severity (Table 5).

**Table 5:** Linear regression of the secondary outcome on blood biomarker measures and covariates.

Independent variables		Delayed Δ in depressive symptom severity*					
Biomarker measures	Covariates	β	n	R^2^	R^2^_adjusted_	RMSE	P Value†
S-100B (µg/l), baseline	Unadjusted	−0.04	24	0.00	−0.04	5.01	0.86
	BDI-II (scores), baseline	−0.39	24	0.02	−0.08	5.09	0.85
	BDI-II (scores), acute Δ	−0.10	24	0.12	0.03	4.83	0.63
	Underwent off-pump procedure	−0.12	24	0.02	−0.07	5.08	0.64
	Urgent priority procedure	−0.07	24	0.05	−0.04	5.01	0.74
	Troponin T at 24 hours (ng/dl)	−0.04	24	0.00	−0.09	5.13	0.88
S-100B (µg/l), total Δ	Unadjusted	0.22	21	0.05	−0.00	4.63	0.35
	S-100B (µg/l), baseline	0.21	21	0.05	−0.05	4.74	0.37
	BDI-II (scores), baseline	0.12	21	0.07	−0.03	4.69	0.64
	BDI-II (scores), acute Δ	0.03	21	0.15	−0.05	4.49	0.90
	Age (years)	0.21	21	0.05	−0.06	4.75	0.38
	Weight (kg)	0.16	21	0.08	−0.02	4.66	0.50
	Body-mass index (kg/m^2^)	0.18	21	0.06	−0.04	4.71	0.46
	β-blocker	0.25	21	0.05	−0.06	4.74	0.25
hs-CRP (mg/l), baseline	Unadjusted	0.28	24	0.08	0.04	4.81	0.18
	BDI-II (scores), baseline	0.29	24	0.10	0.01	4.88	0.18
	BDI-II (scores), acute Δ	0.21	24	0.15	0.07	4.74	0.31
	Charlson comorbidity index	0.29	24	0.08	−0.01	4.93	0.20
	Myocardial infarction†	0.31	24	0.15	0.07	4.74	0.14
	Troponin T at 24 hours (ng/dl)	0.30	24	0.09	−0.00	4.91	0.18
hs-CRP (mg/l), total Δ	Unadjusted	−0.07	20	0.00	−0.05	4.77	0.78
	hs-CRP (mg/l), baseline	−0.07	20	0.00	−0.11	4.91	0.79
	BDI-II (scores), baseline	−0.08	20	0.06	−0.05	4.77	0.73
	BDI-II (scores), acute Δ	0.00	20	0.16	0.06	4.51	0.99
	Age (years)	−0.05	20	0.01	−0.11	4.90	0.85
	Underwent off-pump procedure	−0.10	20	0.01	−0.11	4.91	0.77
	Urgent priority procedure	−0.07	20	0.01	−0.11	4.90	0.78
IL-6 (pg/ml), baseline	Unadjusted	−0.10	24	0.01	−0.04	4.99	0.64
	BDI-II (scores), baseline	−0.15	24	0.04	−0.06	5.04	0.50
	BDI-II (scores), acute Δ	−0.13	24	0.12	0.04	4.81	0.54
	Myocardial infarction‡	−0.13	24	0.07	−0.02	4.96	0.54
	Unstable angina at admission	−0.25	24	0.08	−0.01	4.94	0.32
IL-6 (pg/ml), total Δ	Unadjusted	0.16	22	0.03	−0.02	4.89	0.47
	IL-6 (pg/ml), baseline	0.17	22	0.03	−0.07	5.01	0.47
	BDI-II (scores), baseline	0.14	22	0.06	−0.04	4.94	0.54
	BDI-II (scores), acute Δ	0.11	22	0.15	0.06	4.69	0.63
	Acetylsalicylic acid‡	0.17	22	0.03	−0.07	5.01	0.46
	Underwent off-pump procedure	0.31	22	0.06	−0.04	4.92	0.28
	Urgent priority procedure	0.15	22	0.04	−0.06	4.97	0.52

BDI-II = Beck Depression Inventory II. *β* = standardized regression coefficient. hs-CRP = high-sensitivity C-reactive protein. IL-6 = interleukin-6. *R^2^* = coefficient of determination. *R^2^*_adjusted_ = adjusted coefficient of determination. RMSE = root-mean-square error. S-100B = S-100 calcium-binding protein B.

*Delayed Δ refers to the secondary outcome, delayed change in depressive symptom severity, defined as the intra-subject difference between BDI-II scores at baseline and six-month follow-up

† Corresponds to the blood biomarker term in the model.

‡ Within 7 days before the index procedure.

## Discussion

This is the first study, to our knowledge, to have prospectively assessed the relationships between blood biomarkers of glial activation and injury, blood–brain barrier disruption, and depressive symptom severity in the context of the systemic inflammatory challenge paradigm of coronary artery bypass grafting. We found that perioperative increases in the glial activation and injury and blood–brain barrier-disruption biomarker, S-100B, exhibited a strong positive correlation with acute changes in depressive symptom severity, largely independent of covariates associated with either. Given the magnitude of the test statistic for this correlation (P = 0.0004), the statistical significance of this finding cannot readily be attributed to the fact that we did multiple pre-specified comparisons that included assessing the relationship between depressive symptoms and two other biomarkers besides S-100B (α/comparisons = [0.05]/[3] = 0.001>0.0004). We also found that baseline BDI-II scores correlated negatively with both perioperative increases in S-100B levels as well as acute changes in depressive symptom severity, and that adjusting for baseline BDI-II scores diminished the strength and statistical significance of their association (the term for perioperative increases in S-100B levels still approached statistical significance at P = 0.064). Adjusting for baseline BDI-II scores also increased the proportion of variance in the primary outcome explained by the model (from 23% to 35%). Exploratory analyses indicated that the association between perioperative increases in S-100B levels and acute changes in depressive severity remained statistically significant after adjusting for baseline BDI-II scores among participants with BDI-II scores greater than or equal to 5 at baseline (Spearman ρ, 0.66; P = 0.0007). This was not the case, however, among those with BDI-II scores less than 5 at baseline (Spearman ρ, 0.15; P = 0.15).

Two earlier studies assessed relationships between depression and inflammation in the context of coronary artery bypass grafting [38], [39]. In the first of these, Yang and colleagues found that mean levels of hs-CRP at baseline were significantly higher among those with clinical depression at six-month follow-up (defined as a Patient Health Questionnaire (PHQ-9) score ≥9) compared with those who were not [38]. By comparison, the correlation between baseline blood levels of hs-CRP blood levels and six-month BDI-II scores in the present study was not statistically significant. A possible explanation for this apparent discrepancy is that six-month follow-up depressive symptom severity was defined as a dichotomous outcome in Yang and colleagues’ study, whereas it was defined as a continuous outcome in the present study. Supporting this interpretation, results of an exploratory analysis we performed showed that baseline hs-CRP blood levels were significantly higher among participants who were clinically depressed at six-month follow-up (defined as a BDI-II score ≥14), as compared with participants who were not (26.9±41.1 mg/l (*n* = 4) vs 5.6±6.3 mg/l (*n* = 21); mean difference, 21.3 mg/l; 95% confidence interval, 3.3 to 39.3 mg/l; P = 0.022). The same between-group comparison for interleukin-6 blood levels at baseline approached statistical significance (16.0±15.4 pg/ml (*n* = 4) vs 7.0±7.9 pg/ml (*n* = 21); mean difference, 8.5 pg/ml; 95% confidence interval, 1.4 to 19.4 pg/ml; P = 0.09). In the second study, Poole and colleagues found that elevated hs-CRP levels at postoperative day four mediated the association between depression and an increased length of stay [39]. Differences in the aims and designs of this study compared with the present one limit the ability to directly compare and contrast their findings.

The present study has limitations. First, some studies have shown that cardiopulmonary bypass can produce a transient elevation in blood S-100B levels, potentially confounding the observed correlation between perioperative increases in S-100B levels and acute changes in depressive symptom severity [101]. However, subsequent studies, which performed serial assays of blood S-100B levels among patients undergoing coronary artery bypass grafting found that increases in blood S-100B levels due to cardiopulmonary bypass are no longer apparent after 24 hours [102], [103]. Accordingly, because we collected postoperative blood samples at about 48 hours, it is unlikely that perioperative increases in S-100B levels in our study were related to cardiopulmonary bypass. Supporting this interpretation, perioperative increases in S-100B levels among participants in our study did not differ significantly between patients who had cardiopulmonary bypass and those who did not. Second, whether the observed perioperative increases in blood S-100B levels are reliably attributable to glial activation and injury and blood-brain barrier disruption rather than non-cardiac extracerebral sources remains subject to interpretation. This concern arises primarily in the context of two animal model studies having shown a disconnect between S-100B levels in blood and those in CSF or brain parenchyma [68], and one recent cross-sectional study that found a negative correlation between CSF levels of S-100B and depressive symptom severity among patients with non-inflammatory neurological disorders [62]. Arguing in the opposite direction, however, other studies have shown that extracerebral sources do not influence blood S-100B levels [104]. In addition, a recent study of patients undergoing coronary artery bypass grafting found that S-100B levels in both blood and CSF increased together with those of the quintessential astroglial marker glial fibrillary acidic protein as well as CSF-to-serum albumin ratios [81]. Last, since data on antidepressant and statin use were not collected for the original Scarecrow trial, the effect of these drugs on the observed correlation between changes in S-100B levels and acute changes in depressive symptom severity cannot be excluded.

In conclusion, perioperative increases in S-100B levels exhibited a strongly positive correlation with acute changes in depressive symptom severity in the systemic inflammatory challenge paradigm of cardiac surgery. There were no significant associations between either acute or delayed changes in depressive symptom severity and baseline S-100B levels, baseline hs-CRP or interleukin-6 levels, or perioperative changes in hs-CRP or interleukin-6 levels. Among most participants, levels of all three biomarkers were normal at baseline and markedly elevated at 2 days postoperative. Taken together, these findings are consistent with the hypothesis that depression can be mechanistically linked to glial activation and injury as well as blood-brain barrier disruption in the context of systemic inflammation challenge paradigms, to include cardiac surgery. Large prospective cohort studies that can inform the reproducibility of these findings are warranted.
